# Diagnosing Chronic Obstructive Airway Disease on a Smartphone Using Patient-Reported Symptoms and Cough Analysis: Diagnostic Accuracy Study

**DOI:** 10.2196/24587

**Published:** 2020-11-10

**Authors:** Paul Porter, Scott Claxton, Joanna Brisbane, Natasha Bear, Javan Wood, Vesa Peltonen, Phillip Della, Fiona Purdie, Claire Smith, Udantha Abeyratne

**Affiliations:** 1 Joondalup Health Campus Perth Australia; 2 School of Nursing, Midwifery and Paramedicine Curtin University Perth Australia; 3 Partnering in Health Innovations Research Group Joondalup Health Campus Perth Australia; 4 Genesis Care Sleep and Respiratory Perth Australia; 5 Bear Statistics Perth Australia; 6 ResApp Health Brisbane Australia; 7 School of Information Technology and Electrical Engineering University of Queensland Brisbane Australia

**Keywords:** respiratory, medicine, diagnostic algorithm, telehealth, acute care

## Abstract

**Background:**

Rapid and accurate diagnosis of chronic obstructive pulmonary disease (COPD) is problematic in acute care settings, particularly in the presence of infective comorbidities.

**Objective:**

The aim of this study was to develop a rapid smartphone-based algorithm for the detection of COPD in the presence or absence of acute respiratory infection and evaluate diagnostic accuracy on an independent validation set.

**Methods:**

Participants aged 40 to 75 years with or without symptoms of respiratory disease who had no chronic respiratory condition apart from COPD, chronic bronchitis, or emphysema were recruited into the study. The algorithm analyzed 5 cough sounds and 4 patient-reported clinical symptoms, providing a diagnosis in less than 1 minute. Clinical diagnoses were determined by a specialist physician using all available case notes, including spirometry where available.

**Results:**

The algorithm demonstrated high positive percent agreement (PPA) and negative percent agreement (NPA) with clinical diagnosis for COPD in the total cohort (N=252; PPA=93.8%, NPA=77.0%, area under the curve [AUC]=0.95), in participants with pneumonia or infective exacerbations of COPD (n=117; PPA=86.7%, NPA=80.5%, AUC=0.93), and in participants without an infective comorbidity (n=135; PPA=100.0%, NPA=74.0%, AUC=0.97). In those who had their COPD confirmed by spirometry (n=229), PPA was 100.0% and NPA was 77.0%, with an AUC of 0.97.

**Conclusions:**

The algorithm demonstrated high agreement with clinical diagnosis and rapidly detected COPD in participants presenting with or without other infective lung illnesses. The algorithm can be installed on a smartphone to provide bedside diagnosis of COPD in acute care settings, inform treatment regimens, and identify those at increased risk of mortality due to seasonal or other respiratory ailments.

**Trial Registration:**

Australian New Zealand Clinical Trials Registry ACTRN12618001521213; http://www.anzctr.org.au/Trial/Registration/TrialReview.aspx?id=375939

## Introduction

Chronic obstructive pulmonary disease (COPD) is the fourth leading cause of mortality, affecting more than 384 million individuals worldwide [1]. It is characterized by airflow limitation and a progressive decline in lung function [2]. The population prevalence of COPD via spirometry screening is reported to be 9% to 26% in those older than 40 years [3]. It is estimated that 80% of people with COPD are undiagnosed [4], and up to 60% of those with a diagnosis of COPD have been found to be misdiagnosed upon subsequent spirometry [5,6]. Moreover, 30% to 60% of patients who have been diagnosed by a physician as having COPD have not undergone spirometry testing [7]. In a study of 533 patients with COPD, 15% of those with spirometry tests did not show obstruction and 45% did not fulfill quality criteria [8].

COPD should be considered in patients who present with dyspnea, chronic cough, sputum production, or recurrent lower respiratory tract infections and patients who have been exposed to tobacco or air pollution. Airflow limitation, demonstrated by a forced expiratory volume in the first second to forced vital capacity (FEV_1_/FVC) ratio of <0.7 on postbronchodilator spirometry, is considered diagnostic of COPD according to criteria stipulated by the Global Initiative for Chronic Obstructive Lung Disease (GOLD) [2]. The severity of airflow limitation in COPD can be classified by the degree of reduction in FEV_1_ as a percentage of the predicted value [2]. However, spirometry is not routinely used in emergency departments or primary care settings due to inexperience, time constraints, and availability of equipment [9]. Further, the COPD remote patient monitoring equipment (spirometers and oximeters) with the most technological promise and compatibility with daily living are expensive and of limited use [10].

Early and accurate diagnosis of COPD is imperative to ensure initiation of correct treatment, particularly as evidence suggests that the incipient stages represent a period of rapid decline in lung function, during which cessation of smoking and targeted intervention may be of value [11]. Rapid identification and management of COPD is important in acute care settings, as there is a heightened risk of mortality from respiratory infections such as seasonal influenza [12]. SARS-CoV-2 has a reported case fatality rate of 1.4% for patients without comorbid conditions versus 8.0% for those with chronic respiratory conditions [13].

Screening for COPD in primary care settings using spirometry in asymptomatic patients has not been found to be efficient, as high numbers of patients need to be screened to detect any cases [14,15]. Screening questionnaires, such as the COPD diagnostic questionnaire (CDQ), have performed poorly in an asymptomatic cohort in the primary care setting [16]. We propose that the best use of an algorithm for screening is in a scenario in which patients present to a health care facility with symptoms, as this has a higher pretest probability of case detection.

We previously demonstrated high diagnostic agreement of an automated algorithm with clinical diagnoses for pediatric respiratory diseases, including croup, asthma, bronchiolitis, and pneumonia. The algorithm also accurately separated upper from lower respiratory tract conditions [17]. The technology, which has regulatory approval, is similar to that used in speech recognition software and combines lower airway audio data transmitted during cough events and simple patient-reported clinical symptoms to derive the diagnostic probability output [18]. As the lower airway is open to the outside during a cough, sounds are transmitted through the mouth and can be recorded. In this way, it is similar to traditional auscultation; however, much higher bandwidth is achievable using our method, as the chest wall no longer reduces sound transmission. We recorded audio using a standard smartphone, and the built-in diagnostic algorithm provided a rapid result without requiring clinical examination or additional diagnostic tests.

In this paper, we describe the development and evaluate the accuracy of an algorithm for diagnosing COPD in a cohort of mixed respiratory disorders, including acute respiratory infections. The intended use population is patients who present to health settings with suspected respiratory illness.

## Methods

### Study Population and Setting

Between January 2016 and March 2019, a convenience study sample was obtained by prospectively recruiting participants from the emergency department, low-acuity ambulatory care, and inpatient wards of a large general hospital in Western Australia and from the consulting rooms of a respiratory physician.

This diagnostic accuracy study is part of a more extensive development program (Breathe Easy; Australian New Zealand Clinical Trials Registry ACTRN12618001521213). Patients were approached if they presented to a participating site with signs or symptoms of respiratory disease or to specialist rooms for a lung function test. Patients with no discernible symptoms of respiratory disease were also recruited. Patients were excluded if they were on ventilatory support, had a terminal disease, were medically unstable, had structural upper airway disease, or had a medical contraindication to providing a voluntary cough (eg, severe respiratory distress; eye, chest, or abdominal surgery within 3 months; history of pneumothorax). Patients with uncontrolled heart failure or cardiomyopathy, neuromuscular disease, or lobectomy or pneumonectomy were also excluded. From this cohort, only those aged 40 to 75 years were enrolled in the COPD development program.

Written informed consent was obtained from all participants, and the study was approved by a human research ethics committee (Reference No. 1501). There were no adverse events reported. The study did not interfere with clinical care and all treatment decisions were at the discretion of the treating physician.

### Index Test (Software Algorithm)

The development of the mathematical techniques used to derive the algorithm has been described in depth elsewhere [17-20]. Briefly, an independent training cohort (N=564) was used to obtain clinical data and cough samples (from which mathematical features were extracted). In developing the algorithm, selected features were weighted and combined to build various continuous classifier models used to determine the probability of a COPD diagnosis (reference test). The probability output of the algorithm represents the specific, weighted combination of features. Multiple clinical symptoms and audio characteristics were examined and combined, with the goal to minimize the number of inputs and to use patient-reported symptoms rather than clinically determined signs, vital signs, or investigations. Each input added to the overall accuracy and discriminatory clinical ability of the algorithm. The optimal model and corresponding probability decision threshold were selected using a receiver operating characteristic (ROC) curve, with due consideration given to achieving a balance of positive percent agreement (PPA) and negative percent agreement (NPA) [18]. Once the optimal model was developed, it was locked from further development and prospectively tested for accuracy on an independent testing set.

Audio data were obtained from 5 coughs using a smartphone (iPhone 6; Apple Inc) held approximately 50 cm away from the participant at a 45° angle to the direction of the airflow. Recordings were undertaken in standard clinical environments; however, we took care to avoid other people’s coughs and voices. The cough recording was obtained within 30 minutes of the physical examination of the patient to ensure the clinical features had not changed. If the participant was unable to provide 5 coughs that were recognized by the cough detection software or if the cough recording became corrupted, the participant was excluded from further analysis.

The following 4 clinical symptoms were selected for inclusion in the tested model: participant age, smoking pack-years, and participant-reported presence of acute cough or fever during this illness. One smoking pack-year was defined as 20 cigarettes or 20 g of tobacco smoked each day over 1 year [21]. Where the clinical symptoms were partially unknown, the algorithm did not return a response.

### Reference Test (Clinical Diagnosis or Spirometry)

A full medical assessment was performed on all participants at the time of enrollment, including history and clinical examination. Diagnostic tests were ordered by the treating clinician independently of the study and results were available to researchers.

A specialist physician assigned a clinical diagnosis to each participant based on a review of their medical file, including discharge diagnosis, all outpatient and inpatient notations, and radiology and laboratory results. The same clinical diagnosis definitions (Table 1) were employed in both the testing set (described here) and in the training set used for algorithm development.

Spirometry was performed according to standard methodology [2,22].

**Table 1:** Clinical diagnosis definitions.

Condition	Definition
COPD^a^	Respiratory symptoms consistent with COPD and history of smoking (>10 pack-years) or environmental exposure AND: If spirometry performed, then FEV_1_/FVC^b^ <0.7 on the best test (after bronchodilator) OR If spirometry not performed, then previous physician diagnosis of COPD
COPD (infectious exacerbation)	ALL OF: Met COPD case definition (as above) Worsening symptoms of SOB^c^ or cough Signs and symptoms of acute respiratory tract infection
Acute LRTI^d^	New lower respiratory tract symptoms (SOB, cough, chest pain <1 week) and acute fever AND: For pneumonia: new consolidation on CXR^e^ or CT^f^ OR For LRTI: infiltrate but no consolidation on CXR or CXR not performed
No lower airway disease	No lung disease and spirometry results within normal parameters (FEV_1_/FVC >0.7 on best test)

^a^COPD: chronic obstructive pulmonary disease.

^b^FEV_1_/FVC: forced expiratory volume in the first second to forced vital capacity.

^c^SOB: shortness of breath.

^d^LRTI: lower respiratory tract infection.

^e^CXR: chest x-ray.

^f^CT: computed tomography.

### Analysis Population

Diagnostic accuracy tests were performed for 4 groups using an independent test set of participants. The same inclusion and exclusion criteria were used for both training and test sets (Table 2).

After a clinical diagnosis was assigned to all participants, the database was locked and the software algorithm was run by an independent researcher to ensure blinding was maintained. Each participant’s cough sound data and clinical diagnosis were only used once in the prospective test.

**Table 2:** Analysis groups.

Group name	Role	Participants included and excluded
Group 1: COPD^a^ total cohort^b^	To determine the presence or absence of COPD	Included participants with: COPD with and without acute lower respiratory tract infections (pneumonia and LRTI^c^) Chronic bronchitis, emphysema, or chronic asthma (with and without acute lower respiratory tract infections, such as pneumonia and LRTI) No underlying COPD with acute lower respiratory tract infections (pneumonia and LRTI) No lower airway disease Excluded participants with physician-diagnosed episodic asthma who were experiencing an isolated acute exacerbation or physician-diagnosed restrictive lung disease
Group 2A: COPD with infectious comorbidity	To determine the presence or absence of COPD when participants with COPD also have an acute LRTI	All of group 1, excluding participants with COPD without LRTI
Group 2B: COPD without infectious comorbidity	To determine the presence or absence of COPD when participants with COPD do not have an acute LRTI	All of group 1, excluding participants with COPD with LRTI
Group 3: COPD confirmed by spirometry	To determine the presence or absence of spirometry-confirmed COPD	Of group 1, excluding those whose COPD was not confirmed by spirometry

^a^COPD: chronic obstructive pulmonary disease.

^b^From the total cohort (group 1), groups 2A, 2B, and 3 were derived.

^c^LRTI: lower respiratory tract infection.

### Statistical Analysis

Power calculations were derived as follows. Based on an expected positive and negative percent agreement greater than 85% from the training program, to obtain a superiority end point of 75% (lower bound 95% confidence interval of maximum width within 0.10), a minimum of 48 cases were required.

PPA is defined as the percentage of participants with a positive index test result for a specified condition who also have a positive reference standard for the same condition. NPA is the percentage of participants who return negative results for both tests.

The primary study end point was defined as the PPA and the NPA of the index test with the reference standard, with 95% confidence intervals calculated using the Clopper-Pearson method. The probability of positive clinical diagnosis was calculated for each participant by the final classifier model and was used as the decision threshold in the derived ROC curve.

## Results

In the prospective testing set, 270 participants met inclusion criteria for and were enrolled in the COPD diagnostic study. Of these, 153 were from the hospital emergency department or inpatient wards, and 117 were respiratory outpatients or from the ambulatory acute care unit.

**Figure 1:**
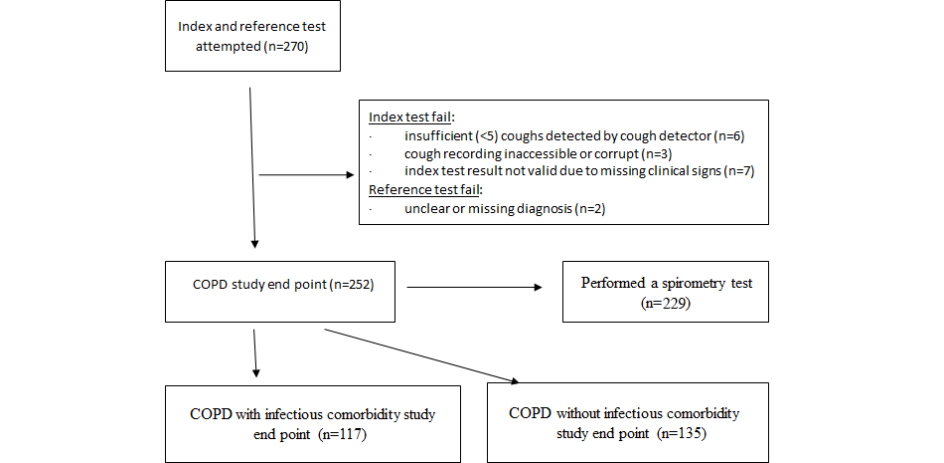
The flow of participants through the study. COPD: chronic pulmonary obstructive disease.

A total of 252 participants provided a valid index and reference test (Figure 1); 2 were excluded because the clinical diagnosis was recorded as unsure. The mean age of the participants was 59.7 (SD 9.2) years, and 148 of the 252 (58.7%) participants were women. Those with COPD were older than those without COPD (65.5 vs 57.8 years; *P*<.001), although the sex proportion did not differ with the diagnosis. Of the 252 participants analyzed, 215 (85.3%) had at least one of the following respiratory symptoms: acute, chronic, or productive cough; fever; rhinorrhea; shortness of breath; wheeze; or hoarse voice. Participant characteristics are shown in Table 3, including spirometry results where available.

**Table 3:** Participant characteristics. Data include all participants in analyzed groups (COPD positive and negative).

Characteristic		COPD^a^ total cohort (group 1, N=252)	COPD with infectious comorbidity (group 2A, n=117)	COPD without infectious comorbidity (group 2B, n=135)	COPD confirmed by spirometry (group 3, n=229)
Age, mean (SD)		59.7 (9.2)	60.6 (9.1)	59.0 (9.1)	59.0 (9.1)
BMI, mean (SD)		28.8 (7.3)	29.0 (7.9)	28.6 (6.7)	29.2 (7.3)
FEV_1_^b^, mean (SD)		2.3 (1.0)	0.9 (0.2)	2.3 (1.0)	2.3 (1.0)
FVC^c^, mean (SD)		3.2 (1.1)	1.9 (0.4)	3.3 (1.1)	3.2 (1.1)
FEV_1_/FVC, mean (SD)		69.1 (16.2)	46.1 (11.3)	70.5 (15.4)	69.1 (16.2)
Predicted FEV_1_, mean (SD)		81.2 (28.8)	34.3 (12.3)	84.0 (27.0)	81.2 (28.8)
Predicted FVC, mean (SD)		90.7 (22.2)	57.6 (14.0)	92.7 (21.0)	90.7 (22.2)
Predicted FEV_1_/FVC, mean (SD)		83.2 (21.9)	58.4 (14.0)	85.3 (21.1)	83.2 (21.9)
**Acute cough, n (%)**					
	No	136 (54.0)	22 (18.8)	114 (84.4)	129 (56.3)
	Yes	116 (46.0)	95 (81.2)	21 (15.6)	100 (43.7)
**Fever, n (%)**					
	No	126 (58.1)	39 (33.3)	87 (87.0)	114 (58.8)
	Yes	91 (41.9)	78 (66.7)	13 (13.0)	80 (41.2)
**Rhinorrhea, n (%)**					
	No	116 (53.7)	61 (52.1)	55 (55.6)	101 (52.3)
	Yes	100 (46.3)	56 (47.9)	44 (44.4)	92 (47.7)
**Wheeze, n (%)**					
	No	145 (66.8)	84 (71.8)	61 (61.0)	134 (69.1)
	Yes	72 (33.2)	33 (28.2)	39 (39.0)	60 (30.9)

^a^COPD: chronic obstructive pulmonary disease.

^b^FEV_1_: forced expiratory volume in the first second.

^c^FVC: forced vital capacity.

For cases where spirometry (n=229) was used to confirm the presence or absence of COPD, the mean age of participants was 59.0 (SD 9.1) years and 80 (65.0%) participants were women, with FEV_1_ measurements as shown in Table 4. The COPD-negative group included 6 patients with chronic fixed asthma who had an FEV_1_ below 80%.

**Table 4:** Spirometry-derived FEV_1_ (GOLD severity categories) in participants with and without COPD [2].

Percent predicted FEV_1_^a^ (GOLD^b^ severity category)	COPD^c^ positive, n (%)	COPD negative, n (%)
<30.0% (GOLD 4: very severe)	5 (12)	0 (0.0)
30.0% to 49.9% (GOLD 3: severe)	17 (40)	2 (2)
50.0% to 79.9% (GOLD 2: moderate)	16 (38)	4 (5)
≥80.0% (GOLD 1: mild)	4 (10)	75 (93)
Total	42 (100)	81 (100)

^a^FEV_1_: forced expiratory volume in the first second.

^b^GOLD: Global Initiative for Chronic Obstructive Lung Disease.

^c^COPD: chronic obstructive pulmonary disease.

**Figure 2:**
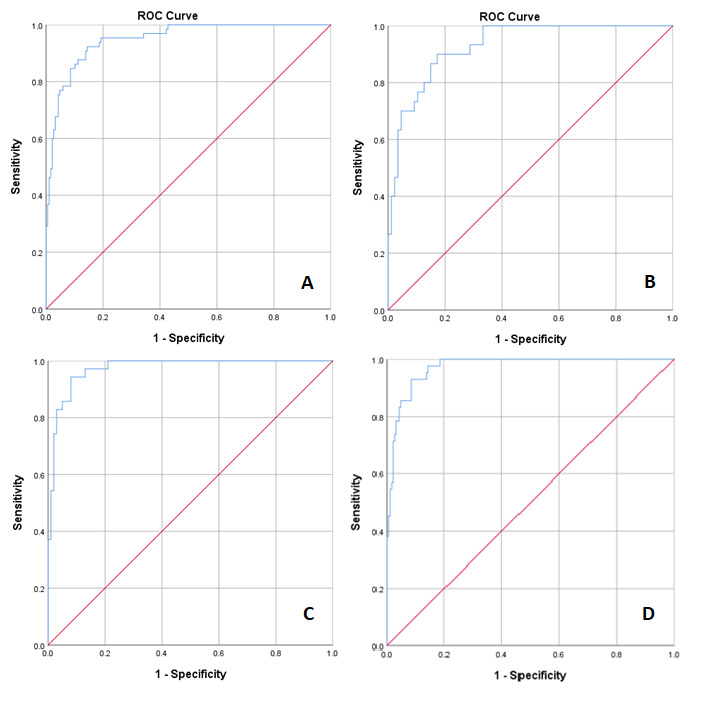
Receiver operating characteristic curve and AUC for (A) COPD total cohort (group 1), AUC=0.95 (95% CI 0.92-0.98); (B) COPD with infectious comorbidity (group 2A), AUC=0.93 (95% CI 0.88-0.98); (C) COPD without infectious comorbidity (group 2B), AUC=0.974 (95% CI 0.95-1.00); (D) COPD diagnosed by spirometry group (group 3), AUC=0.973 (95% CI 0.95-1.00). AUC: area under the curve; COPD: chronic obstructive pulmonary disease.

The calculated PPA and NPA of the algorithm with clinical diagnosis and area under the curve (AUC) are shown in Table 5. ROC curves for each test group are shown in Figure 2.

Although the algorithm was developed to discriminate based on GOLD criteria, we repeated the analysis using lower limit of normal (LLN) thresholds to diagnose COPD. Test performance in the COPD confirmed by spirometry group (n=229) returned a PPA of 100% (95% CI 90.75%-100.0%) and an NPA of 75.4% (95% CI 68.65%-81.32%).

**Table 5:** PPA, NPA, and calculated AUC of the algorithm (index test) compared with clinical diagnosis (reference test).

Group	PPA^a^, % (95% CI); n/N	NPA^b^, % (95% CI); n/N	AUC^c^ (95% CI)
Group 1: COPD^d^ total cohort (n=252)	93.8 (85.0-98.3); 61/65	77.0 (70.3-82.8); 144/187	0.95 (0.9-1.0)
Group 2A: COPD with infectious comorbidity	86.7 (69.3-96.2); 26/30	80.5 (70.6-88.2); 70/87	0.93 (0.9-1.0)
Group 2B: COPD without infectious comorbidity	100 (90.0-100.0); 35/35	74.0 (64.3-82.3); 74/100	0.97 (0.9-1.0)
Group 3: COPD confirmed by spirometry	100 (91.6-100.0); 42/42	77.0 (70.3-82.8); 144/187	0.97 (0.9-1.0)

^a^PPA: positive percent agreement.

^b^NPA: negative percent agreement.

^c^AUC: area under the curve.

^d^COPD: chronic obstructive pulmonary disease.

## Discussion

We have described a simple, rapid diagnostic test for COPD that demonstrates high agreement with clinical diagnosis in the acute setting. Diagnostic agreement of the software algorithm with clinical diagnosis of COPD showed a PPA of 93.8% and an NPA of 77.0%. Agreement was maintained when the patient had an acute respiratory infection (PPA of 86.7% and NPA of 80.5%). Notably, the index test retained high diagnostic agreement in cases of spirometry-confirmed COPD (PPA of 100.0% and NPA of 77.0%).

Population and primary care surveys have demonstrated that mild (FEV_1_ ≥80% of percent predicted) and moderate (FEV_1_ 50%-80% of percent predicted) airflow limitation is seldom diagnosed by clinicians [23,24]. In our study, 20 out of 42 (48%) participants with clinically diagnosed COPD had only mild or moderate airflow limitation (Table 4). This group represents those who would benefit most from this algorithm, both because of new treatment possibilities and because they are frequently underdiagnosed.

We used the GOLD criteria for COPD diagnosis (FEV_1_/FVC <0.7) when developing our algorithms, although COPD can also be defined using the LLN. When calculated using the LLN thresholds, test performance was not significantly different from values obtained using the GOLD criteria. It should be noted that, as our model was developed to recognize COPD diagnosed using the GOLD criteria, we would expect a lower performance when the diagnostic criteria are changed.

In many European countries, spirometry is available in acute and primary care settings [8]. However, uptake of the test is limited, leading to underdiagnosis or misdiagnosis of patients [6]. Several barriers to using spirometry in primary and acute care settings have been reported, including expense and limitations in access, expertise, and time [25]. Alternative testing methods have been developed. A meta-analysis of the CDQ in ever-smokers in 4 studies had a pooled sensitivity of 64.5% (95% CI 59.9%-68.8%) and a specificity of 65.2% (52.9%-75.8%) [16]. Another study recruiting current and former smokers over 40 years from the general population demonstrated moderate sensitivity and specificity of the CDQ (74% and 72%, respectively), the COPD Population Screener (56% and 90%, respectively), and the Lung Function Questionnaire (79% and 68%, respectively) [26]. An analysis from 3 studies of handheld flow meters showed a sensitivity of 79.9% (95% CI 74.2%-84.7%) and a specificity of 84.4% (95% CI 68.9%-93.0%) [16]. In a scenario comparable to our study, when the CDQ was performed on symptomatic patients in primary care, the AUC was 0.65, sensitivity was 89.2% and 65.8%, and specificity was 24.4% and 54.0% for participants at low risk and high risk of having COPD, respectively [27]. The performance of our software algorithm exceeds that of the currently available COPD screening questionnaires, outperforms the sensitivity of handheld flow meters with comparable specificity, and demonstrates high agreement with the gold standard (spirometry) in under one minute. This algorithm is intended to be used as a stand-alone device, allowing for real-time diagnosis. As it is easy to operate and requires no physical patient contact, infection risk is minimized.

We envisage that the algorithm could be used as an initial screening test in acute care settings for patients who present with nonspecific respiratory symptoms. A positive result could be used to guide immediate care in the acute setting. As the test is delivered via smartphone, it could be applied in person or during a telehealth consultation. A formal diagnosis of COPD requires confirmation by spirometry, the gold standard tool for COPD diagnosis [2]. Confirmatory spirometry could be performed during subsequent specialist follow-up.

In this study, we were able to accurately identify the presence or absence of COPD in patients with lower respiratory tract infections, including pneumonia. In these situations, spirometry can be difficult to perform adequately, and an initial diagnostic test will help detect COPD in acutely unwell patients and identify those individuals most at risk of developing complications. Individuals with COPD are known to experience more frequent complications and higher mortality rates due to seasonal illnesses such as influenza [12]. More recently, a meta-analysis examining the risk of severe outcomes from SARS-CoV-2 infection (admission to the intensive care unit, mechanical ventilation, or death) showed a greater than fivefold increase in the risk of severe disease in patients with coexistent COPD [28]. We recommend that all patients with COPD with a suspected infection be carefully monitored in view of this increased risk. The diagnosis of COPD in patients presenting with SARS-CoV-2 or similar respiratory infections would allow more focused therapeutic pathways and guide health care resources to this at-risk group.

There are several limitations to this study. Our study population was recruited in an urban setting and had smoking-related COPD. The generalizability of these results to COPD of differing etiologies and in other settings requires confirmation. The tests were performed by trained research personnel in controlled environments, although we would consider the device less onerous to use than spirometry. The cough recording can be affected by background noise and positioning of the device, although the program will alert the user if background noise is excessive. The population recruited reflects the intended age range of use. However, as expected, those with diagnosed COPD were slightly older than those without COPD, and it will be important to replicate this study using an older control group.

The COPD diagnostic algorithm described in this study is used in combination with a suite of other respiratory diagnostic algorithms developed in the Breathe Easy program, including tests for asthma, pneumonia, and lower respiratory tract disease [17]. The software provides a diagnostic output for each condition simultaneously every time it is used. Having independent decision algorithms for asthma, COPD, and pneumonia is particularly important due to the considerable clinical overlap between the conditions.

In conclusion, the algorithm was able to accurately identify COPD, even in the presence of infection. The algorithm operates as a stand-alone tool and provides a rapid result. It may find application in the acute care setting as a screening tool to alert clinicians to the presence of COPD, allowing for more rapid, targeted, and appropriate management.

## Abbreviations

AUC: area under the curve
CDQ: chronic obstructive pulmonary disease diagnostic questionnaire
COPD: chronic obstructive pulmonary disease
FEV_1_: forced expiratory volume in the first second
FVC: forced vital capacity
GOLD: Global Initiative for Chronic Obstructive Lung Disease
LLN: lower limit of normal
NPA: negative percent agreement
PPA: positive percent agreement
ROC: receiver operating characteristic
